# Emphysema distribution and annual changes in pulmonary function in male patients with chronic obstructive pulmonary disease

**DOI:** 10.1186/1465-9921-13-31

**Published:** 2012-04-18

**Authors:** Naoya Tanabe, Shigeo Muro, Shiro Tanaka, Susumu Sato, Tsuyoshi Oguma, Hirofumi Kiyokawa, Tamaki Takahashi, Daisuke Kinose, Yuma Hoshino, Takeshi Kubo, Emiko Ogawa, Toyohiro Hirai, Michiaki Mishima

**Affiliations:** 1 Department of Respiratory Medicine, Graduate School of Medicine, Kyoto University, 53 Kawahara-cho, Shogoin, Sakyo-ku, Kyoto, 606-8507, Japan; 2 Division of Clinical Trial Design and Management, Translational Research Center, Kyoto University Hospital, Kyoto, Japan; 3 Department of Diagnostic Imaging and Nuclear Medicine, Kyoto University, Kyoto, Japan

**Keywords:** Chronic obstructive pulmonary diseases, COPD, CT, Emphysema, Lung function, Heterogeneity

## Abstract

**Background:**

The progression of chronic obstructive pulmonary disease (COPD) considerably varies among patients. Those with emphysema identified by quantitative computed tomography (CT) are associated with the rapid progression assessed by forced expiratory volume in one second (FEV_1_). However, whether the rate of the decline in lung function is independently affected by the regional distribution or the severity of emphysema in the whole lung is unclear.

**Methods:**

We followed up 131 male patients with COPD for a median of 3.7 years. We measured wall area percent (WA%) in right apical segmental bronchus, total lung volume, percent low attenuation volume (LAV%), and the standard deviation (SD) of LAV% values from CT images of 10 isovolumetric partitions (SD-LAV) as an index of cranial-caudal emphysema heterogeneity. Annual changes in FEV_1_ were then determined using a random coefficient model and relative contribution of baseline clinical parameters, pulmonary function, and CT indexes including LAV%, SD-LAV, and WA% to annual changes in FEV_1_ were examined.

**Results:**

The mean (SD) annual change in FEV_1_ was −44.4 (10.8) mL. Multivariate random coefficient model showed that higher baseline FEV_1_, higher LAV%, current smoking, and lower SD-LAV independently contributed to an excessive decline in FEV_1_, whereas ratio of residual volume to total lung capacity, ratio of diffusing capacity to alveolar ventilation, and WA% did not, after adjusting for age, height, weight, and ratio of CT-measured total lung volume to physiologically-measured total lung capacity.

**Conclusions:**

A more homogeneous distribution of emphysema contributed to an accelerated decline in FEV_1_ independently of baseline pulmonary function, whole-lung emphysema severity, and smoking status. In addition to whole-lung analysis of emphysema, CT assessment of the cranial-caudal distribution of emphysema might be useful for predicting rapid, progressive disease and for developing a targeted strategy with which to prevent disease progression.

## Background

Chronic obstructive pulmonary disease (COPD) is a leading cause of death that is characterized by airflow limitation [1]. Forced expiratory volume in one second (FEV_1_) is central to assess airflow limitations and diagnose COPD, and the decline in FEV_1_ is one of the most important outcome measures for evaluating disease progression in longitudinal observational studies and clinical trials [2-5]. On the other hand, the clinical manifestation of COPD is rather heterogeneous in terms of imaging and disease progression [6,7]. Changes in FEV_1_ might also vary among patients [4,7,8].

Identifying subgroups with different prognostic or therapeutic characteristics as a specific phenotype is important to improve COPD management [6]. Several reports have shown that quantitative computed tomography (CT) is useful for phenotyping patients with COPD because it provides structural information about emphysematous change, airway dimensions and lung volume [9-11]. Especially, since emphysema and airway disease are two major features of COPD and the relative involvement of these two factors considerably vary among patients with COPD, the utility of CT-based categorization (emphysema-predominant, airway-predominant, or mixed) is being increasingly recognized [12-15]. Indeed, it has been shown that CT findings of emphysema, but not airway wall thickening correlate with low body mass index [13], and that both CT measures of emphysema and airway disease are associated with respiratory symptoms and frequency of COPD exacerbations [14,16]. We previously demonstrated that the extent of emphysema assessed by CT predicts mortality in patients with COPD independently of FEV_1_[17]. Moreover, the decline in FEV_1_ is greater in patients with, than without the emphysema phenotype [4] and emphysema severity is associated with a rapid annual decline in FEV_1_[8]. These findings indicate that the CT-based emphysema phenotype provides information when monitoring patients with COPD.

In addition to the whole lung quantitation of emphysema, CT imaging can quantify the distribution of emphysematous change [18-20]. Quantitative CT studies have shown that the rates of homogeneous emphysema distribution and of lower lung-predominant emphysema in patients with COPD are not as small as estimated from visual assessment [20,21]. Upper lung-dominant emphysema is associated with genetic variants in MMP-9 [22] or EPHX1 and GSTP1 [23] and the cranial-caudal emphysema distribution is a powerful predictor of the response to lung volume reduction surgery [24]. Moreover, lower lung-dominant emphysema can predict mortality in patients with emphysema independently of emphysematous changes in the whole lung [25]. These findings suggest that the cranial-caudal heterogeneity of emphysema can be used to further categorize patients with the emphysema phenotype. However, whether regional emphysema heterogeneity affects the rate of disease progression remains unclear.

The present study investigates whether the cranial-caudal heterogeneity of emphysema affects the decline in FEV_1_ independently of the total amount of emphysematous change. We followed up 131 male patients with COPD for a median period of 3.7 years, and annual changes in FEV_1_ were calculated using a random coefficient model. We then investigated which factors at study entry can contribute to the decline in FEV_1_.

## Methods

### Study design and study population

**Figure 1:**
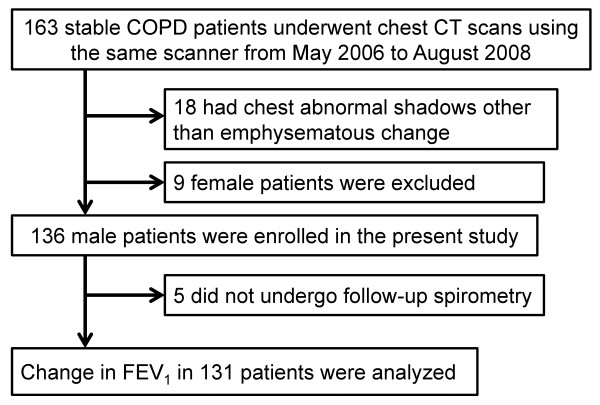
Protocol of the present study.

The protocol of this investigation, which was part of an ongoing COPD observational study at Kyoto University, is summarized in Figure 1. Some patients had also participated in our previous studies [10,26,27]. We recruited 163 patients with stable COPD between March 2006 and August 2008 from an outpatient clinic at Kyoto University Hospital. Exclusion criteria comprised a history of chest disease other than COPD, refusal to undergo CT scanning or a diagnosis of α1-antitrypsin deficiency. We excluded 18 patients with abnormal shadows not associated with emphysematous changes in chest CT images at study entry. We also excluded female patients because the number of female patients (n = 9) was too small to determine the impact of sex on pulmonary function decline. Thus, 136 male patients who were examined by spirometry every six months were followed up until July 2011. The rate of decline in lung function was analyzed in a dataset of patients in whom FEV_1_ was measured at least twice as described below. The ethics committee of Kyoto University approved the study (approval No. E182) and all patients provided written informed consent to participate.

### Clinical and pulmonary function examinations

During exacerbation-free periods, the patients inhaled short-acting bronchodilators and then underwent pulmonary function tests (Chestac-65 V; Chest MI Corp; Tokyo, Japan) and high resolution CT at full inspiration (Aquilion 64; Toshiba; Tokyo, Japan; 0.5-mm slice thickness) as described [10,28]. Lung volumes and diffusion capacity were measured by helium dilution and the single-breath method, respectively. The use of only one CT scanner avoided inter-scanner variability, and CT parameters were analysed using a custom-designed application [10]. We defined exacerbation of COPD as symptomatic deterioration requiring antibiotics and/or systemic corticosteroid. In patients who had been followed up at Kyoto University Hospital during the year before study entry, exacerbations were counted.

### Analysis of CT images

We measured total lung volume, the ratio of low attenuation volume to the lung volume (LAV%) in the whole lung with −960 HU as the cut-off level, and wall area percent (WA%) in the right apical segmental bronchus as described [9,10,28,29]. By modifying the reported method [16,30], we estimated the level of inspiration during CT scanning. The ratio of CT-derived total lung volume to physiologically-measured total lung capacity (TLC) was calculated as a proxy for inspiration level, and the proxy was used for statistical analysis. Total lung volume was divided into equal upper and lower regions, and the difference in LAV% between the two regions was calculated as described [18,25]. Positive and negative values for differences represent the upper and lower dominance of emphysema, respectively.

**Figure 2:**
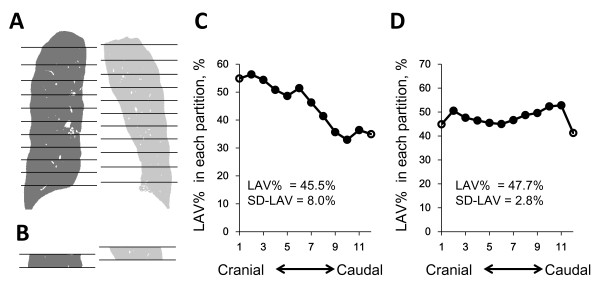
**Illustration of the methods to evaluate the cranial-caudal heterogeneity of emphysematous change.** Each lung was divided into 12 partitions with equal volumes (**A**), and LAV% values in 12 partitions were calculated (**B**). The top and basal partitions (open circles in **C** and **D**) were excluded to avoid volume partial effects. SD-LAV was defined as the standard deviation of LAV% values in the remaining 10 partitions. **C** and **D** showed examples of heterogeneous and homogeneous emphysema, respectively. While LAV% in C and D were almost similar (45.5 and 47.7%, respectively), SD-LAV in C was higher than in D (8.0 versus 2.8%).

To assess the cranial-caudal heterogeneity of emphysema, we calculated standard deviation (SD) of values of LAV% in 10 partitions with equal volume (SD-LAV) using a modification of the reported method [19] as follows. As shown in Figure 2, each lung was divided into 12 equal volumes from the top to the base, and the most apical and basal partitions were excluded to eliminate the volume partial effect. Low attenuation volume was measured in each partition. After summing low attenuation volumes of identical partitions in the right and left lungs, LAV% in each partition was measured by dividing the summed value by the volume of the partition. Then, the SD of LAV% values in the 10 partitions was calculated to obtain SD-LAV. Higher and lower SD-LAV represent the more heterogeneous and homogeneous distribution of emphysema, respectively.

### Statistical analyses

Data are expressed as means ± SD. Individual annual changes in FEV_1_ were determined from empirical Bayes estimates using a random coefficient model with a linear term of time and random effects of intercepts and slopes for each patient. We assessed the relative contributions of LAV% and SD-LAV to the annual decline in FEV_1_ using a random coefficient model that included baseline age, height, weight, percent predicted FEV_1_ (%FEV_1_), ratio of residual volume to TLC (RV/TLC), ratio of diffusing capacity to alveolar ventilation (D_LCO_/V_A_), smoking status, LAV%, SD-LAV, WA%, and the proxy for inspiration, and the interaction of each covariate with time. A history of frequent exacerbations (≥ 2 exacerbations per year) was not included in the analysis of all the enrolled patients, because 12 of them had not been followed up in the previous year, and they were included only in a supplementary analysis. All *p-*values are two-sided and *p* < 0.05 was considered significant. All data were statistically analysed using JMP 7 software (SAS Institute, Cary, NC, USA).

## Results

### Characteristics of patients

Figure 1 shows that we excluded 5 of the 136 enrolled patients without follow-up spirometry. The median follow-up period was 3.7 years, and detailed information about follow-up spirometry, the follow-up period, use of regular COPD medication and clinical course are provided in Supplementary Table E1, E2, and E3 ( Additional file 1). We found that of 119 (91%) patients whose history of exacerbations was recorded for one year before study entry, 15 had experienced at least two exacerbations.

The baseline characteristics of patients and CT parameters are shown in Table 1. SD-LAV% significantly correlated with the absolute value of the difference in LAV% between the upper and lower lung regions (r = 0.94, *p* < 0.001), indicating that a higher SD-LAV% was associated with more upper or lower predominant emphysema.

**Table 1:** Baseline characteristics of study patients (n = 131)

	
Age (y)	70.7 ± 8.8
Height (cm)	164.4 ± 6.1
Weight (kg)	58.1 ± 8.9
Smoking status (current: former)	34 : 97
Smoking history (pack-years)	67.7 ± 34.1
Pulmonary function	
FEV_1_ (L)	1.63 ± 0.68
%FEV_1_ (%)	57.9 ± 19.8
RV/TLC (%)	43.3 ± 8.1
D_LCO_/V_A_ (mL/min/mmHg/L)	2.77 ± 1.08
CT parameters	
LAV% (%)	33.0 ± 8.6
Total lung volume (L)	5.38 ± 0.9
Proxy for inspiration	0.93 ± 0.13
WA (right apical bronchus %)	57.8 ± 5.8
SD-LAV (%)	4.1 ± 2.5
Difference in LAV% (upper – lower lung)	
≥ 0% (upper predominant)	n = 94
<0% (lower predominant)	n = 37

Data are expressed as means ± SD.

Abbreviations: FEV_1_, forced expiratory volume in one second;

%FEV_1_, FEV_1_% predicted; RV/TLC, ratio of residual volume to total lung capacity;

D_LCO_/V_A_, ratio of diffusing capacity to alveolar ventilation; LAV%, ratio of

low attenuation volume to total lung volume; WA%, wall area percent in right apical bronchus;

Proxy for inspiration was defined as the ratio of CT-derived total lung volume

to physiologically measured total lung capacity;

SD-LAV, standard deviation of LAV% values in 10 partitions with equal volumes.

### Annual change in FEV_1_ and baseline quantitative CT measurements

**Figure 3:**
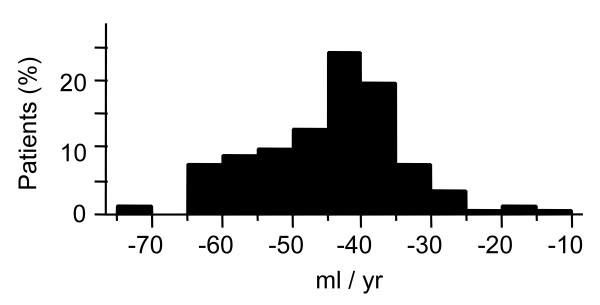
**A histogram of annual change in post-bronchodilator FEV**_**1.**_

The mean (SD) annual change in FEV_1_ was −44.4 (10.8) mL (Figure 3). The multivariate random coefficient model showed that a higher %FEV_1_, current smoking, higher LAV% in the whole lung, and lower SD-LAV independently contributed to the annual decline in FEV_1_, whereas RV/TLC, D_LCO_/V_A_, and WA% did not, after adjustment for age, height, weight, and the proxy for inspiration (Table 2). The findings of this analysis revealed that a 1% increase in each of %FEV_1_ and LAV%, and a 1% decrease in SD-LAV contributed to an additional decline of 0.97, 2.46, and 3.24 mL, respectively. The annual decline in FEV_1_ was 20.90 mL greater in current, than in former smokers. Our analysis of 119 (91%) patients showed that SD-LAV remained independently associated with changes in FEV_1_, whereas a history of frequent exacerbations did not (Table 3).

**Table 2:** **Prognostic factors contributing to annual change in FEV**_**1 **_**in multivariate random coefficient model (n = 131) **

	Estimate*	**95% Confidence interval**	***p*-value**
Age, per 1-year increase	0.86	(0.10 to 1.63)	0.028
Height, per increase of 1 cm	0.03	(−1.11 to 1.17)	0.96
Weight, per increase of 1 kg	0.03	(−0.84 to 0.90)	0.95
%FEV_1_, per 1% increase	−0.97	(−1.51 to −0.44)	<0.0001
RV/TLC (%)	0.03	(−1.06 to 1.13)	0.95
D_LCO_/V_A_ (mL/min/mmHg/L)	−0.65	(−8.83 to 7.52)	0.88
Continuous smoking, yes/no	−20.90	(−37.24 to −4.57)	0.013
LAV%, per 1% increase	−2.46	(−3.81 to −1.10)	<0.0001
SD-LAV, per 1% increase	3.24	(0.82 to 5.87)	0.016
WA%, per 1% increase	−0.63	(−1.67 to 0.41)	0.23
Proxy for inspiration^†^, per increase of 0.1	−1.67	(−7.36 to 4.01)	0.56

Abbreviations: %FEV_1_, percent predicted FEV_1_; RV/TLC, ratio of residual volume to total lung capacity;

D_LCO_/V_A_, ratio of diffusing capacity to alveolar ventilation; LAV%, ratio of low attenuation volume to total lung volume; SD-LAV, standard deviation of LAV% values in 10 partitions with equal volumes;

WA%, wall area percent in right apical bronchus.

^†^Proxy for inspiration was defined as the ratio of CT-derived total lung volume to physiologically measured total lung capacity for estimating inspiration levels during CT scan.

*Regression coefficients for interactions with time, a negative value indicates excess annual decline in FEV_1._.

**Table 3:** **Prognostic factors contributing to annual changes in FEV**_**1 **_**in multivariate random coefficient model: Sub-analysis of 119 patients whose history of exacerbations was recorded for one year before study entry**

	Estimate*	**95% Confidence interval**	***p*-value**
Age, per 1-year increase	0.88	(0.04 to 1.71)	0.040
Height, per increase of 1 cm	0.13	(−1.06 to 1.31)	0.83
Weight, per increase of 1 kg	−0.09	(−1.00 to 0.81)	0.84
%FEV_1_, per 1% increase	−0.87	(−1.44 to −0.31)	0.003
RV/TLC (%)	0.03	(−1.11 to 1.18)	0.95
D_LCO_/V_A_ (mL/min/mmHg/L)	−1.50	(−10.48 to 7.48)	0.74
Continuous smoking, yes/no	−22.76	(−40.13 to −5.40)	0.011
LAV%, per 1% increase	−2.62	(−4.04 to −1.20)	0.0004
SD-LAV, per 1% increase	3.32	(0.54 to 6.10)	0.020
WA%, per 1% increase	−0.48	(−1.56 to 0.60)	0.26
Proxy for inspiration^†^, per increase of 0.1	−0.79	(−6.76 to 5.18)	0.79
History of frequent exacerbation in previous year, yes/no	5.91	(−13.10 to 24.91)	0.54

Frequent exacerbation is defined as at least two exacerbations per year.

Abbreviations: %FEV_1_, percent predicted FEV_1_; RV/TLC, ratio of residual volume to total lung capacity; D_LCO_/V_A_, ratio of diffusing capacity to alveolar ventilation; LAV%, ratio of low attenuation volume to total lung volume; SD-LAV, standard deviation of LAV% values in 10 partitions with equal volumes; WA%, wall area percent in right apical bronchus;

^†^Proxy for inspiration was defined as the ratio of CT derived total lung volume to physiologically-measured total lung capacity for estimating inspiration levels during CT scan.

* Regression coefficients for interactions with time, negative value indicates excess annual decline in FEV_1_.

## Discussion

The present study found that in addition to LAV%, smoking status, and baseline %FEV_1_, cranial-caudal emphysema heterogeneity (assessed by SD-LAV) were independently associated with an annual decline in lung function in male patients with COPD. The CT-based emphysema phenotype is associated with a rapid decline in FEV_1_ and the severity of emphysema increases the risk for a rapid decline [4,8]. However, as far as we can ascertain, this is the first study to prospectively demonstrate that a more homogeneous distribution of emphysema contributes to an accelerated decline in FEV_1_ independently of the severity of emphysema.

The progression of COPD is heterogeneous [4,6-8]. Exploring risk factors for disease progression and establishing the appropriate phenotype are important to design a targeted strategy for preventing disease progression. Radiographic phenotyping by CT has considerable potential [6,31], because it provides structural information that might reflect clinical manifestations independently of conventional lung function [14,16]. We previously showed that the severity of emphysematous change predicts COPD mortality independently of lung function [17]. The present study found that a higher LAV% and a lower SD-LAV contributed to an accelerated decline in lung function independently of baseline %FEV_1_.

In addition to SD-LAV, we measured the difference in LAV% between the upper and lower lung regions, and found rather frequent lower predominant emphysema in patients without an α1-antitrypsin deficiency. This is consistent with previous reports, in which smokers might not always have upper predominant emphysema [20,21]. We considered that susceptibility to a rapid decline in lung function is lower in patients with upper or lower predominant, than homogeneous emphysema.

The determinants of emphysema distribution are not fully understood, but genetic and environmental factors are thought to be involved. Previous studies have shown that variants of MMP-9 [22] or EPHX1 and GSTP1 [23] are associated with upper-predominant emphysema. We speculate that a currently unrecognized genetic background is associated with susceptibility to homogenous emphysema, and with a rapid decline in lung function. A comparison of clinical manifestations or genetic backgrounds between homogeneous and heterogeneous emphysema should increase understanding of the factors associated with accelerated disease progression.

Airway dimensions were also analyzed because the potential of CT-based categorization into emphysema- and airway-dominant phenotypes has been suggested [12-14]. Unlike LAV%, WA% in the proximal airway was not associated with the annual decline in FEV_1_, although proximal airway dimensions correlate with respiratory symptoms, health-related quality of life and exacerbation frequency [14,16,32]. We postulated that the rapid decline in FEV_1_ is impacted more by the emphysema- than the airway-dominant phenotype.

The present study showed that the mean annual change in FEV_1_ was −44.4 mL. This is consistent with the findings of two large clinical trials, in which the annual decline in FEV_1_ was about 40 mL in the treatment groups [3,5,33]. On the other hand, two recent observational studies found a relatively modest decline of 32 to 33 mL per year [4,8]. Some COPD medications such as tiotropium and salmeterol/fluticasone combination might reduce the decline in FEV_1_[33,34], and the present study reconfirmed that the baseline severity of airflow limitation is inversely related to the decline in FEV_1_.[4,7,33] We speculated that a difference in the baseline characteristics could account for small discrepancies among different studies.

The present study reconfirmed the deleterious effect of smoking and the importance of smoking cessation. Current smoking caused an additional 20.9 mL per year decline in FEV_1_ (Table 2). This is consistent with findings of other studies [4,35], in which the rate of decline was 20–30 mL per year greater in current, than in former smokers.

Exacerbation of COPD can cause a rapid decline in FEV_1_[4,33,36,37]. We therefore performed a sub-analysis (Table 3), which included a history of frequent exacerbations that can identify patients with exacerbation susceptibility [38]. We found that SD-LAV remained independently associated with a decline in FEV_1_. However, the impact of a history of frequent exacerbations was not detected. It have been shown that exacerbations during follow-up are associated with an additional decline in FEV_1_, but the number of exacerbations in the previous year cannot predict the future decline in FEV_1_[4,33]. Thus, to further clarify the relative contribution of the emphysema distribution and exacerbations to the decline in FEV_1_, a model including exacerbations during follow-up should be analyzed in future studies.

D_LCO_/V_A_ and RV/TLC were also included in the present random coefficient models. Tables 2 and 3 show that baseline D_LCO_/V_A_ and RV/TLC were not associated with the subsequent change in FEV_1_. The lack of the association of D_LCO_/V_A_ with the decline in FEV_1_ in the model including LAV% might have been because D_LCO_/V_A_ correlates well with LAV%. Indeed, when we constructed the other random coefficient model including the same valuables except LAV%, lower D_LCO_/V_A_ tended to contribute to the decline in FEV_1_ (p = 0.051).

Since inappropriate inspiration during CT scanning can affect lung density, CT-derived total lung volume has been used to adjust for the variability of the inspiration level [16,39]. Previous studies have estimated the inspiration level by assessing the ratio of CT-derived total lung to predicted TLC [16,30]. We modified this method in the present study. The ratio of CT-derived total lung volume to TLC obtained by lung function tests was calculated as the proxy for inspiration level, and then the proxy was included into the multivariate random coefficient model to lessen this confounding effect.

As shown in Supplementary Table E1 (Additional file 1), the present random coefficient model included 14 (11%) patients whose follow-up periods were less than 2 years. Although patients who were lost to follow-up can be generally included into the random coefficient model, we perform subanalysis of 117 patients who were followed up for at least two years. Consequently, we confirmed that SD-LAV remained independently associated with annual decline in FEV_1_ (*p* = 0.009)_._

The limitations in the present study include a smaller study population than those found in other studies [3-5]. However, we used the same scanner to increase consistency among CT data and thus overcome the disadvantages of a small sample cohort. This strategy generated valid information about the severity of emphysema and the heterogeneity of cranial-caudal emphysema.

We could not recruit enough female patients to explore the influence of sex on the decline in FEV_1_, and thus excluded female patients in the present study. Since susceptibility to cigarette smoke, the severity and distribution of emphysema, and the extent of airway lesions could differ between male and female patients [30,40,41], the present findings cannot be generalized to female patients with COPD.

The present study is observational, and COPD medications were selected by pulmonologists at Kyoto University according to the GOLD guideline [1]. Some COPD medications such as salmeterol/fluticasone combination and tiotropium might have modified the rate of change in FEV_1_[33,34]. However, we believe that this influence is quite small because the relative contribution of LAV% and SD-LAV to the change in FEV_1_ persisted after adjustment for baseline %FEV_1_ and a history of exacerbations, which could mainly affect the choice of COPD medication.

We measured airway dimensions in the proximal, but not in the distal airways, although the major site of airway obstruction in patients with COPD is small airways [42]. Since proximal airway dimensions assessed by CT predict histological measurements of small-airway wall dimensions [43], we believe that the location of the airways in which we measured WA% impacted the present results minimally or not at all.

## Conclusion

We found that a more homogeneous distribution of emphysema independently contributed to an accelerated decline in lung function in addition to higher baseline lung function, more severe emphysematous changes in the whole lung, and current smoking. This not only strengthens the value of the CT-based emphysema phenotype as a predictor of a rapid decline in lung function, but also provides new information; a more homogeneous distribution of emphysema increases susceptibility to rapid disease progression.

## Abbreviations

COPD: Chronic obstructive pulmonary disease; CT: Computed tomography; DLCO/VA: Ratio of diffusing capacity to alveolar ventilation; FEV1: Forced expiratory volume in one second; %FEV1: Percent predicted FEV1; LAV%: Percent low attenuation volume; Proxy for inspiration: Ratio of CT-derived total lung volume to physiologically-measured total lung capacity; SD-LAV: Standard deviation of values of LAV% in 10 partitions with equal volume (index for cranial-caudal heterogeneity of emphysema); TLC: Total lung capacity; RV/TLC: Ratio of residual volume to total lung capacity; WA%: Wall area percent.

## Competing interests

The authors have no conflicts of interest to disclose.

## Authors’ contributions

NT, SM, ST, SS, TO, HK, TT, DK, YH, TK, EO, TH, and MM contributed to the study design, collection of data, and analysis and interpretation of data. NT and TO contributed to the development of the custom-made application for analyzing CT images. SM and MM contributed to the acquisition of funding. ST contributed to the statistical analysis of data. NT and SM conducted manuscript preparation. All authors have read and approved the final manuscript.

## Supplementary Material

Additional file 1 Supplementary tables for the present study. The number of follow-up spirometry and follow-up period, regular COPD medication during follow-up, and clinical course of study patients are shown in supplementary table E1, E2, and E3, respectively. Click here for file
