# Supplementary material for: Emphysema distribution and annual changes in pulmonary function in male patients with chronic obstructive pulmonary disease
Source: Respir Res. 2012 Apr 18;13(1):31. doi: 10.1186/1465-9921-13-31 (PMC3478996; doi:10.1186/1465-9921-13-31)
Supplement: Additional file 1 — Supplementary tables for the present study. The number of follow-up spirometry and follow-up period, regular COPD medication during follow-up, and clinical course of study patients are shown in supplementary table E1, E2, and E3, respectively. [file 1465-9921-13-31-S1.pdf]

Supplementary Table E1. Follow-up spirometry and follow-up period (n = 131).

Follow-up spirometric measurements	N (%)
1 - 2	9 (7)
3 - 4	28 (21)
5 - 6	55 (42)
6 - 7	38 (29)
8 - 9	1 (1)
Follow-up period (y)	
< 2	14 (11)
2 - 3	9 (7)
3 - 4	50 (38)
4 - 5	51 (39)
≥ 5	7 (5)

Supplementary Table E2. Regular COPD medication during follow-up (n = 131).

Medication	N (%)
Tiotropium	71 (54)
Salmeterol/fluticasone combination	23 (18)
Inhaled corticosteroid	47 (36)
Long-acting ß agonist	32 (24)
Any COPD regular medications (including, tiotropium, salmeterol/fluticasone combination, inhaled corticosteroid, and long-acting β agonist)	94 (72%)

Supplementary Table E3. Clinical course of study patients (n =131).

	N (%)
Ongoing follow-up	106 (81)
Discontinuation of follow-up spirometry	15 (11)
Loss to follow-up	8
Severe respiratory failure	5
Stroke	1
Congestive heart failure	1
Death	10 (8)
Respiratory disease	8
Heart disease	2

Status of patients ascertained at July 2011.
